# Monoclonal Antibodies in Gynecological Cancer: A Critical Point of View

**DOI:** 10.1155/2011/890758

**Published:** 2011-12-26

**Authors:** Filippo Bellati, Chiara Napoletano, Maria Luisa Gasparri, Valeria Visconti, Ilaria Grazia Zizzari, Ilary Ruscito, Jlenia Caccetta, Aurelia Rughetti, Pierluigi Benedetti-Panici, Marianna Nuti

**Affiliations:** ^1^Department of Gynecology and Obstetrics, “Sapienza” University of Rome, Italy; ^2^Department of Experimental Medicine, “Sapienza” University of Rome, Italy

## Abstract

During the last decades, several improvements in treating gynecological malignancies have been achieved. In particular, target therapies, mostly monoclonal antibodies, have emerged as an attractive option for the treatment of these malignancies. In fact, various molecular-targeted agents have been developed for a variety of malignancies with the objective to interfere with a precise tumor associated receptor, essential for cancer cell survival or proliferation, blocking its function, of the cancer cells. Alternatively, monoclonal antibodies have been developed to block immune suppression or enhance functions of immune effector cells. So far, several monoclonal antibodies have been tested for clinical efficacy for the treatment of gynecological cancers. Antibodies against Vascular Endothelial Growth Factor (VEGF) and Epidermal Growth Factor Receptor (EGFR) have been used in different neoplasms such as ovarian and cervical cancer. Catumazumab, a bivalent antibody against CD3 and EpCAM, is effective in the treatment of neoplastic ascites. Other antibodies are peculiar for specific cancer-associated antigen such as Oregovomab against CA125 or Farletuzumab against the folate receptor. Here we describe the preclinical and clinical experience gained up to now with monoclonal antibodies in tumors of the female genital tract and trace future therapeutic and research venues.

## 1. Introduction

Despite the improvement achieved during the last decades in gynecological cancer treatment, most of these patients, especially women affected by ovarian cancer, are at great risk of recurrence and emerging drug resistance. Therefore, novel approaches are required to improve outcomes for gynecological cancer patients. Recently, various molecular-targeted agents have been developed and used in the management of a variety of malignancies, including ovarian, cervical, and endometrial cancers. The therapeutic benefits of targeted clinical interventions, with increased selectivity and fewer adverse effects, hold great promises in the treatment of solid malignancies, both as single therapy and in combination. In particular, Monoclonal Antibodies (MoAbs) represent the majority of target therapies which have been investigated and employed in clinical settings so far. These immunological reagents recognize molecular targets whose expression is tumor associated or/and are essential for the cancer cell survival and proliferation such as the Vascular Endothelial Growth Factor (VEGF), the Epidermal Growth Factor Receptor (EGFR) family, CA125, MUC1, and other signaling pathways which are aberrant in tumor tissue (EpCAM). Also, the targeting of immune cells by MoAbs has been proved to be an efficacious strategy to modulate immune system functions (anti-CTLA-4, anti-CD3, anti-CD40). To date, several MoAbs have been approved for the treatment of colorectal, breast, head and neck, nonsmall cell lung, and renal cell cancer (Table 1). Encouraging results have being achieved also in gynecological tumors. Here, we review the most promising MoAbs that are under early or advanced investigation for the treatment of neoplasms of the lower genital tract.

## 2. Rationale of Monoclonal Antibodies in Cancer Treatment

Significant advances in gynecological cancer management have been recently achieved, including interesting progresses in surgical, chemotherapeutic, and concurrent chemo-radioterapeutic settings. However, more effective, specific, and less toxic approaches need to be investigated. Based on the promising results of preclinical studies, various targeted therapies are currently being evaluated in cancer patients. One of the most promising approaches, that may improve patient outcome, is the use of MoAbs. The use of MoAbs in cancer treatment is focused on the idea of selectively targeting tumor cells that express tumor-associated antigen [1], with the aim to specifically antagonize receptor signaling pathways, which are essential for proliferation, survival, and migration of tumor cells. Thus, MoAbs offer increasingly customized solutions based on the targeting of multiple specific pathways essential for cancer development and metastasis by attacking targeted tumor cells. Furthermore, the high specificity of the target reduces cytotoxic side effects on normal tissue, seen with traditional chemotherapeutic agents, and should permit the maintenance of a high quality of life. The first experience of MoAb administration in cancer patient was carried out in a patient affected by non-Hodgkin's lymphoma [2]. Since then, several MoAbs against cancer-associated antigens have been developed and MoAbs have rapidly become one of the biggest classes of new drugs approved for the treatment of cancer (Table 1). To date, several ongoing trials are investigating the role of MoAbs in ovarian, cervical, and endometrial cancer (Tables 2–5). In some cases, MoAbs have already demonstrated favorable clinical outcomes in phase I/II studies and are being investigated further in phase III trials. However, further investigations for most of these molecules are required to establish a convincing proof of safety and efficacy of them in gynecological tumors.

## 3. Monoclonal Antibodies: Mechanisms of Action

MoAbs are antibodies produced by hybridoma cells. In the sixties the conventional route to derive MoAbs was to immunize mice. It took 10 years to be translated to the patient with MoAb muromonab, a murine-derived antibody for acute organ rejection approved by FDA in 1986 [3]. Recently, recombinant engineering techniques permitted the construction of MoAbs with possible variation in size, valence, configuration, and effectors functions. This technology results in the development of fragment, chimeric, humanized, and fully humanized MoAbs.

MoAb therapy consists in targeting specific extracellular/cell-surface pathways in order to destroy malignant tumor cells and prevent tumor growth by blocking specific cell receptors.

Binding specificity and selective molecular targeting are the major advantages of this approach. The general mechanism mediated by MoAb administration is the specific recognition of an antigen selectively expressed by tumor cells and the generation of immune-complexed cells, that can activate distinct immune mechanisms mainly mediated by the Fc region of the MoAb. Increased uptake by antigen presenting cells, NK activation, and induction of ADCC are the effects described. Moreover, the engineering of the Fc domain permits to increase affinity towards specific FcRs, potentiating the action of specific innate immune cells. This is the case of MoAbs directed against antigens that are homogenously overexpressed by cancer cells such as MUC1 and CA125. The identification of molecules that have a key role in tumor progression and immune modulation has led to the generation of immune reagents that combine specificity to the ability to exert a biological function on the target cell. Three main approaches can be identified.

**(1) MoAbs Recognizing Specific Tumor-Associated Receptors**

Tumor cells display specific receptors that are rare or absent on the surfaces of healthy cells, and which are responsible for activating cellular signal transduction pathways that cause the unregulated growth and division of the tumor cell. Specific targeting of these receptors can block tumor-associated transduction pathways, reducing tumorigenicity and invasiveness. MoAbs, such as trastuzumab, act through this mechanism [4].

**(2) MoAbs Targeting Tumor Promoting Molecules**

During tumor transformation several tumor promoting molecules are produced by the cancer cells, suppressing and subverting the function of immune system. The administration of MoAbs targeting such molecules can interfere in the binding of the molecule to its receptor or/and increases the clearence of these soluble factors, thus reducing tumor cell growth. Bevacizumab, the MoAb against VEGF molecule, is a paradigmatic example. In fact, binding of bevacizumab to VEGF blocks VEGF binding to its receptor [5].

**(3) MoAbs Targeting Immune Effector Cells**

The targeting of immune cells can be achieved by the employment of MoAbs specific for surface receptors that can have suppressing or activating function. The CTLA-4 molecule expressed by effector cells exerts an inhibitory function and the functional blocking of this molecule is being investigated in clinical trials [6].

On the other hand, targeting of activating molecules, such as CD3, is a strategy to enhance the functions of immune effector cells. The strategy utilized is to generate a bispecific antibody able to recognize simultaneously a relevant tumor antigen and an immune-specific activating molecule. In this way, the antibody combines the specific recognition of the target cells to the selective activation of the immune effector cell, bringing the two cells physically close, thus making easier the immune recognition. Catumaxomab, a bispecific antibody recognizing EpCAM and the CD3 molecule, is a prototype of such reagents [6].

## 4. Monaclonal Antibodies in Ovarian Cancer

In contrast to hematological malignancies and certain solid tumors such as breast and colorectal cancer, MoAbs have not been completely proven to be clinically effective in the treatment of ovarian cancer, although encouraging results are being achieving. Currently, the mostly investigated targets in ovarian cancer are (VEGF) and (EGFR) family members (EGFR1, EGFR2/ErbB2).

Other tumor-associated antigens, such as the adhesion molecule EpCAM, the epithelial mucins CA125 and MUC1, and the Folate Receptor as well as molecules expressed by immune cells such as CD3 and CTLA-4 are under evaluation.

### 4.1. Vascular Endothelial Growth Factor-Targeted Therapy: Bevacizumab

#### 4.1.1. Vascular Endothelial Growth Factor (VEGF)

VEGF, also known as Vascular Permeability Factor, is a potent angiogenetic cytokine that induces mitosis and regulates the permeability of endothelial cells.

Overexpression of VEGF correlates with increased microvascular density, cancer recurrence, and decreased survival in several neoplasms, including most gynaecological tumors [7–13].

In women with ovarian cancer, high serum levels of VEGF are found to be an independent risk factor for ascites, advanced-stage disease, undifferentiated histology, number of metastasis, and decreased survival [9–11]. In ovarian carcinoma there is suggestive evidence showing that higher VEGF levels are associated with aggressive clinical behavior.

#### 4.1.2. Bevacizumab

Bevacizumab represents the most investigated target therapy in ovarian neoplasia. It is a humanized monoclonal antibody directed against the VEGF ligand able to inhibit the formation of new blood vessels and to decrease the diameter, density, and permeability of blood vessels, resulting in a normalization of tumor vascularization [14, 15]. Moreover it has been shown that VEGF acts as immunosuppressive factor, contributing to the skewing of the antitumor immune response and the development of immunosuppressive microenvironment [16]. Randomized trials in solid tumors have shown that the addition of bevacizumab to standard chemotherapeutic regimens results in statistically significant improvements in progression-free survival (PFS) and, in some cases, in overall survival (OS) [17–19]. Currently, bevacizumab has not been approved for any malignancy of the female genital tract, although initial encouraging data have been achieved for these types of neoplasms, especially for ovarian cancer. Up to day, several investigators have explored bevacizumab as a single agent or in combination with chemotherapy in the management of ovarian cancer [20]. Both alone or in combination with traditional drugs, it has shown interesting levels of activity and provided clinically meaningful results in patients with recurrent ovarian disease [21–30]. Furthermore, it has also been used as a palliative treatment of symptomatic ascites [21, 23, 31–35]. In one of these kinds of experience [35], immunological analyses after intraperitoneal bevacizumab administration showed a concomitant increase in number and function of CD8+ T effector cells and a decrease of circulating regulatory T cells (Treg) cells, similarly to what observed in ovarian cancer patients undergoing to debulking surgery and radiotherapy [36]. These effects observed on the immune performance of the patient can be due to the pleiotropic function of VEGF that can directly acts as immunosuppressive molecule on the immune microenvironment [16]. The most considerable results concerning the role of bevacizumab on progressive disease in ovarian cancer patients can be derived from recent analysis of two completed randomized phase III trials: the GOG 218 [37] and ICON 7 [38]. These were performed using bevacizumab in newly diagnosed advanced stage ovarian cancer, in association with standard chemotherapy. The GOG 218 [37] is a three-arm placebo controlled trial: 1873 patients have been randomized to iv paclitaxel-carboplatin for 6 cycles with or without bevacizumab in the latter five followed by placebo or additional 48 weeks of maintenance bevacizumab (15 mg/kg every 3 weeks). Preliminary data, initially presented at the 2010 meeting for the American Society of Clinical Oncology (ASCO), showed a significant improvement in PFS in patients treated with concurrent and maintenance bevacizumab, 14.1 months versus 10.3 months in the placebo arm. Relative to arm 1 of the trial, the hazard ratio for first progression in the maintenance arm of the trial was 0.717 (95% CI: 0.625–0.824, *P* < 0.0001). OS data are not yet mature.

 The ICON7 trial [38] is a two-arm, non-placebo controlled trial comparing carboplatin-paclitaxel (6 cycles) versus carboplatin-paclitaxel-bevacizumab (7,5 mg/kg) every three weeks for 6 cycles, followed by 12 cycles of maintenance bevacizumab or disease progression, whichever occurred earlier. Data from this trial were presented at the 2010 meeting of the European Society of Medical Oncology (ESMO). A total of 1528 women were randomized from 263 centers. Compared to the control arm, the hazard ratio for disease progression in the bevacizumab arm was 0.81 (95% CI: 0.70–0.94, *P* < 0.0041).

In the setting of recurrent ovarian cancer, of great importance will be the mature results of AURELIA trial [39] (so far open to accrual), that is investigating the association of bevacizumab with platinum compounds both in platinum sensitive, and in platinum resistant patients.

The timing of bevacizumab administration during platinum-based regimens is believed to be a crucial point in the design of efficacious therapy in patients with recurrent disease.

The two ongoing phase III trials GOG213 [40] and OCEANS [41] take in consideration such parameter. Both trials target patients with recurrent disease: the former plans the administration of carboplatin and paclitaxel with or without bevacizumab in platinum sensitive relapsed OC patients, while in the latter carboplatin and gemcitabine with or without bevacizumab in recurrent disease, respectively.

In conclusion, up to now data arising from phase III trials show benefits in terms of Disease Free Survival (DFS). The benefit of bevacizumab on OS requires to be better investigated. 

### 4.2. Epidermal Growth Factor Receptor Targeted Therapy: Trastuzumab, Cetuximab and Pertuzumab

#### 4.2.1. Epidermal Growth Factor Receptor (EGFR) Family

EGFR family is a receptor family composed of four structurally similar tyrosine kinase receptors, ErbB1/HER1 (commonly referred to as EGFR), ErbB2/HER2 (commonly referred to as HER2), ErbB3/HER3, and ErbB4/HER4 [42]. They are expressed on the apical surface of epithelial cells. After binding with its ligand, the EGFR undergoes dimerization followed by tyrosine autophosphorylation, leading to the activation of EGFR signaling. Activation of downstream signaling pathways is known to mediate a variety of cellular responses, including cancer cell proliferation, survival, motility, and invasion. Moreover, as these receptors are overexpressed in many solid tumors, they have been recognized as promising targets for cancer therapy. Several MoAbs against the extracellular domain of EGFRs have been developed with the peculiar ability to block signaling of the receptor upon binding. Trastuzumab and Pertuzumab, directed against HER2 molecule and cetuximab (directed towards HER1), are in clinical use for several solid cancers. Also they have been evaluated in the framework of treatment regimens for ovarian cancer.

#### 4.2.2. Trastuzumab (Anti-HER2)

Trastuzumab is a humanized MoAb specific for the extracellular domain of HER2 that has been selected for its ability to block HER2 signaling after binding to the receptor HER2 is overexpressed in approximately 30% of breast cancers and is associated with a more severe prognosis [43, 44]. Trastuzumab is currently approved for refractory breast cancers positive HER2/neu either as a single agent or in combination with paclitaxel. To evaluate the therapeutic potential of trastuzumab in ovarian cancer, several preclinical studies have been conducted using HER2-expressing ovarian cancer cells [45, 46].

At least five potential extracellular and intracellular antitumor mechanisms of trastuzumab have been identified in the preclinical setting. These include activation of antibody-dependent cellular cytotoxicity, inhibition of the activatory extracellular domain cleavage, abrogation of intracellular signaling, reduction of angiogenesis, and decreased DNA repair [47]. Recently, also cellular adaptive immune system has been proposed to play a crucial role in trastuzumab clinical efficacy [48]. The overall results of these synergistic effects lead to tumor cell stasis and/or death.

On the basis of the promising results obtained in breast cancer patients [49] and the results of preclinical studies in ovarian cancer models [45, 46], the first phase II study evaluating the efficacy of trastuzumab in patients with recurrent ovarian cancers overexpressing HER2 was carried out in 2003 [50]. Forty-one women affected by recurrent or refractory ovarian or primary peritoneal carcinoma with 2+ or 3+ HER2 overexpression were enrolled. Patients without progressive disease or grade 3-4 toxicities could continue the treatment indefinitely. Patients with stable or responding disease were offered, after 8 weeks of treatment, to increase the weekly dose up to 4 mg/kg until disease progression. Median treatment duration was 8 weeks (range 2 to 104 weeks) and median progression-free interval was 2 months. Patients were analysed for the presence of soluble extracellular domain of HER2 and antibodies against trastuzumab. Circulating extracellular domain of HER2 increased during treatment in 8 of 24 evaluable patients. This immunological outcome was not associated to clinical outcome. No increase of anti-trastuzumab antibodies was observed. Although trastuzumab was well tolerated with common side effects of anemia, gastrointestinal disturbance, neuropathy, and fatigue, the overall response rate in these patients was only 7% with a median progression-free interval of 2 months. Interestingly, among patients with recurrent ovarian cancer who were screened for participation in the trial, only 11.4% were judged to have overexpression of HER2. On the basis of these results, the GOG was unable to recommend trastuzumab in OC.

#### 4.2.3. Pertuzumab (Anti-HER2)

Pertuzumab is a recombinant, humanized monoclonal antibody binding to the HER2 dimerization domain, sterically blocking the binding pocket required for receptor dimerization with its partner receptors, thus inhibiting the signaling cascades [51]. Pertuzumab binding to HER2 induces activation of ADCC effects but does not block the truncation of HER2 in the same way as trastuzumab binding does [52]. In a phase II study of 123 recurrent ovarian cancer patients, 55 patients in cohort 1 and 62 in cohort 2 were evaluable for efficacy [53]. The patients in cohort 1 received a loading dose of 840 mg of pertuzumab intravenously followed by 420 mg every 3 wk; the patients in cohort 2 received 1050 mg every 3 wk and showed an overall response rate of 4.3%. The main adverse events observed were diarrhea and asymptomatic left ventricular ejection fraction decreases of <50%.

Combination therapy of pertuzumab with gemcitabine was tested in a randomized phase II trial in 130 patients with platinum-resistant ovarian, fallopian tube, or primary peritoneal cancer [54]. The patients were randomly assigned to gemcitabine (800 mg/m^2^ on days 1 and 8 of a 21-day cycle) plus either placebo or pertuzumab (840 mg loading dose followed by 420 mg every 3 wks) and showed objective response rates of 13.8% and 4.6%, respectively. Therefore, pertuzumab was able to significantly increase the effect of gemcitabine.

#### 4.2.4. Cetuximab (Anti-HER1)

Cetuximab is a chimeric MoAb that binds to the extracellular domain of EGFR (HER1).

It was developed to target the EGFR, thus preventing ligand activation of EGFR [55, 56]. In preclinical studies, cetuximab has been able to repress the growth of cultured A431 tumour cells and xenografts that expressed high levels of EGFR [57, 58]. In other solid tumours, cetuximab has shown to enhance the effects of different chemotherapeutic agents, including platinum [59, 60].

Based on these data, cetuximab was administered in combination with carboplatin to 28 patients with relapsed platinum-sensitive ovarian cancer. Cetuximab was infused at an initial dose of 400 mg/m^2^ on cycle 1, day 1, followed by weekly infusions of 250 mg/m^2^. Carboplatin (AUC 6) was administered IV on day 1 at 3-week intervals. The treatment was continued until disease progression or prohibiting toxicities. Twenty-six (92.9%) out of 28 patients were found to have EGFR+ tumours, whereas the remnant 2 patients (7.1%) had EGFR-tumours. Clinical response was reported for EGFR+ tumours: in 9 patients (34.6%) a clinical response was observed (3 (11.5%) Complete Response (CR); 6 (23%) Partial Response (PR)). Three patients (11.5%) had a progressive disease and the remaining 8 patients (30.8%) showed stable disease. The median PFS was over 9.4 months. Some grade 3 and three grade 4 toxicities were experienced but only three could be attributed to cetuximab.

In the same year, cetuximab combined with paclitaxel plus carboplatin was experimented as initial treatment in 40 advanced-stage ovarian, primary peritoneal, or fallopian tube cancer patients [61]. Thirty-eight out of the 40 participants had previously undergone abdominal surgery, whereas the remaining two patients were approached with neoadjuvant chemotherapy. The administration schedule consisted in an initial dose of cetuximab 400 mg/m^2^ IV, followed by weekly infusions of cetuximab 250 mg/m^2^, plus Paclitaxel 175 mg/m^2^ and carboplatin (AUC 6) administered IV at 3-week intervals. Patients obtaining a complete clinical response after 6 cycles were eligible for a maintenance treatment with weekly cetuximab, for 6 months or until progressive disease or major toxicity. Thirty out of 40 patients completed all six cycles of chemotherapy and were evaluable for response: 21 of them achieved a complete clinical response. Twenty patients entered the cetuximab maintenance phase, but only ten completed all six cycles of cetuximab. Ten patients discontinued because of toxicity (5), progressive disease (2), grade 3-sinusitis (1), fluid accumulation (1), or other (1). The overall median time of PFS in the initial population was 14.4 months, with a third of the population with progressive disease after 24 months. Eleven (27.5%) out of 40 patients experienced at least one adverse event to cetuximab, with one case of grade 3-4 toxicity, whereas seven patients (17.5%) experienced toxicity to paclitaxel (three grade 3-4 toxicity). Consequently, although the combination of cetuximab, paclitaxel, and carboplatin was well tolerated in this patient population, this study showed that this combination therapy failed to demonstrate a prolongation of PFS when compared with historical data. In a GOG phase II trial, single agent cetuximab demonstrated only minimal activity in patients with recurrent ovarian cancer with a response rate of 6.3% [62]. GOG has also evaluated the efficacy of cetuximab in the setting of combination therapy with carboplatin in patients with platinum-sensitive recurrent ovarian cancer. Results of this trial showed only modest activity with a response rate of 34.5% [56].

Considering these results, further efforts need to be carried out in the direction of identifying markers that can predict response before cetuximab can become point of the standard treatment.

### 4.3. EpCAM-Targeted Therapy: Catumaxomab

Catumaxomab is the first drug to be approved specifically for the treatment of malignant ascites, thus becoming one of the most successful monoclonal antibody to be employed in oncology. The approval dates back to April 2009, when the European Commission followed the recommendation of the Committee for Human Medicinal Products (CHMP) and approved catumaxomab for the i.p. treatment of malignant ascites in patients with EpCAM+ carcinomas resistant to standard treatments.

Catumaxomab (anti-EpCAM and anti-CD3) is a trifunctional monoclonal antibody with two different specificities, which binds simultaneously to the EpCAM on tumour cells and the CD3-antigen on T-cells. In addition, its Fc region composed by the two Ig isotypes mouse IgG_2a_ and rat IgG_2b_ selectively binds to human Fc*γ*I and III-receptors on innate immune cells, such as macrophages, dendritic cells, and NKs [63, 64]. Ertumaxomab (anti-HER2 x anti-CD3) is another trifunctional monoclonal antibody differing from catumaxomab only because it binds to HER2 rather than EpCAM [65].

Catumaxomab and ertumaxomab were firstly administered intraperitoneally to eight patients with malignant ascites (two of which with ovarian cancer) with the aim of verifying their tolerability and biological and clinical effects [29]. The two ovarian cancer patients were treated with both catumaxomab and ertumaxomab at different administration schedule. The first ovarian cancer patient received six administrations (five with catumaxomab and two with ertumaxomab) during a 13-day period, whereas the other patients were treated with five immunizations (five with ertumaxomab only and the last one in combination with catumaxomab). The treatment was well tolerated by all the eight patients enrolled. Resolution of ascites was experienced by all participants and seven out of eight participants did not require further paracentesis during the follow-up, with a median ascites-free interval of 38 weeks. As expected, the resolution of ascites was correlated with elimination of tumour cells (*P* < 0.0014) as detected by FACS analysis and immunocytochemistry. Complete elimination of EpCAM and HER-2/neu tumour cells in ascites was obtained for both ovarian cancer patients, that succumbed after 22 and 41 weeks, respectively. 

Catumaxomab was also tested in a phase I/II dose-escalating study on 23 women affected by advanced ovarian cancer with symptomatic malignant ascites containing EpCAM+ tumour cells [63]. The participants were divided into six different groups and treated with four to five intraperitoneal catumaxomab in dose 5 to 200 *μ*g on days 0, 3, 6, 9, and 13. All patients were evaluated for toxicity, clinical response, and immunological status. Serious adverse events were detected in 15 out of 23 patients. In six patients they were considered treatment-related. In the majority of participants, a significant decrease of ascites flow rate was observed after the third infusion, compared to baseline. Twenty-two out of 23 patients did not require further paracentesis after the last infusion, until the end of the study at day 37. The results of the prospective randomized phase II/III study published by Heiss et al. in 2010 [66] confirmed the efficacy of catumaxomab in the management of malignant ascites. Two hundred and fifty-eight patients affected by EpCAM+ epithelial tumor-related malignant ascites (129 recurrent ovarian cancer) were randomly assigned to receive paracentesis followed by four i.p. infusion of catumaxomab, in a ten-day period, or paracentesis alone. The Intention to-Treat (ITT) analysis revealed that puncture-free survival was significantly longer in the catumaxomab group than control group (46 versus 11 days; *P* < 0.0001), as well as the median time required for the next paracentesis (77 versus 13 days; *P* < 0.0001). A positive trend in OS was observed in the whole catumaxomab group and in the ovarian cancer patients catumaxomab subgroup; furthermore a significant increase in OS was observed in the gastric cancer patients catumaxomab subgroup. Catumaxomab-related adverse events were manageable, reversible, and associated to an acceptable safety profile.

However, positive results were not reached by catumaxomab treatment in terms of tumor response. In fact, the Phase IIa Study of the AGO Study Group revealed that catumaxomab has modest activity in platinum-resistant ovarian cancer, with only 5% of partial response being obtained with high-dose catumaxumab [67]. 

### 4.4. Folate Receptor Alpha-Targeted Therapy: Farletuzumab

Farletuzumab is a humanized MoAb with high affinity for folate receptor *α* (FR*α*). This receptor, almost absent in normal tissue, is overexpressed in most ovarian cancers, making it an attractive therapeutic target.

 Preclinical studies have demonstrated that farletuzumab mediates robust antibody-dependent cellular cytotoxicity and complement-dependent cytotoxicity *in vitro*, inhibits tumor growth in ovarian tumor xenografts, and displays a safe toxicology profile in not human primates [68, 69].

Farletuzumab has shown clinical efficacy in early phase trials as single agent and combination therapy with minimal drug-specific toxicity [70].

The Phase III development plan in ovarian cancer patients includes combination chemotherapy studies in both platinum-sensitive (recently launched) and platinum-resistant (planned) recurrent disease.

### 4.5. CA125-Targeted Therapy: Oregovomab

CA125 is a surface mucin-like glycoprotein antigen that is expressed in more than 95% of all not mucinous stage III/IV epithelial ovarian cancers (EOCs) [71].

Serum CA125 level is a highly useful and well-established surrogate for monitoring the response to treatment and a useful marker during follow-up [72, 73].

 Oregovomab is a MoAb against the tumour-associated antigen CA125 as both membrane bound and soluble forms. Oregovomab administration induces both cellular and humoral multiepitope immune responses against the tumor cells [74]. Based on the observation that ovarian cancer patients, injected with this agent for diagnostic purpose, showed prolonged survival [75], in 1998 the immunological effects of oregovomab were tested in 75 ovarian cancer patients [76]. All participants received from one to ten injections of the MoAb and, after vaccinations, 64% of them developed anti-idiotypic antibodies against oregovomab, whereas 24% developed anti-CA125 antibodies. It was observed that these two types of antibody were able to induce Fc-mediated tumour cell killing. Moreover, a higher significant survival was observed in patients in which anti-CA125 antibody concentration increased more than 3-fold after oregovomab administrations, compared to patients without such increase. Furthermore, an improved overall survival was observed also in patients who developed specific anti-CA125 B- and T-cell response after oregovomab administration [77]. In 2004, Gordon et al. [78] vaccinated women suffering from recurrent ovarian cancer with oregovomab. Significant increases in T-cell responses were measured in 7/18 (39%) patients in response to CA125, in 5/8 (63%) patients in response to autologous tumor cells, and in 9/18 (50%) patients in response to oregovomab. Immune responses appeared by week 12 (four doses) and were generally maintained or augmented in patients maintaining combined treatment with oregovomab and chemotherapy. Median survival was 70.4 weeks (4.6–141.6 weeks), and the median progression-free interval was 11 weeks (2.6–114.6 weeks). Patients who mounted a T-cell response to CA125 and/or autologous tumor showed significantly improved survival compared to patients who did not.

In 2004, Berek et al. [79] enrolled 145 patients affected by advanced ovarian cancer (FIGO stage III-IV) in a randomized placebo-controlled study, to assess safety, feasibility, and toxicity of oregovomab administration and to evaluate this reagent as consolidation treatment. Unfortunately, despite a benign safety profile, Time to Relapse (TTR) was not significantly improved by consolidation therapy with oregovomab (13.3 months oregovomab versus 10.3 months for placebo; *P* = 0.71).

One year later, Ehlen et al. [80] showed immune and clinical results of a pilot phase 2 study concerning oregovomab-based vaccination in 13 patients with recurrent ovarian cancer. Immune responses, including antibodies and T cells to oregovomab and CA125, were demonstrated in more than half of the patients. Disease stabilization and survival >2 years was observed in 3 of 13 patients and coincided with robust immune responses. Shrinkage of marker lesions was not observed; however, four patients showed decreases in CA125 levels. Treatment was well tolerated without serious adverse events. This pilot study supported immunologic activity and safety of oregovomab in recurrent OC.

Long-term clinical results of this study were showed in 2008 [81], after a 5-year follow-up. Patients assigned to the oregovomab and placebo groups lived a median of 57 and 48.6 months of progression-free survival (*P* = 0.276), respectively. Considering the time of survival after relapse, oregovomab and placebo groups lived 31.2 and 20.7 months, respectively. Further analyses from this study were recently reported [82]. A total of 371 patients were included in the study: 251 were treated with oregovomab and 120 were assigned to the placebo group. After five years, 169 and 80 patients belonging to the oregovomab and placebo group, respectively, were still on treatment. It was observed that the median time to relapse was 10.3 months for oregovomab and 12.9 months for placebo-treated patients, respectively. Survival data were not available at the time of the report. The incidence of treatment adverse events was similar in both groups. These data indicate that patients with advanced ovarian cancer do not benefit from oregovomab maintenance monoimmunotherpy.

 In 2009, a study assessing this immunotherapy at 2 dosing schedules in 40 patients with advanced ovarian cancer undergoing front-line carboplatin-paclitaxel chemotherapy showed that combination of oregovomab immunotherapy and chemotherapy exerted immune adjuvant properties. The possible combination of carboplatin and paclitaxel chemotherapy with oregovomab and other antigen-specific cancer immunotherapy approaches should be further investigated.

Results obtained so far indicate that although CA125 remains an attractive target for immunotherapy, no effective clinical benefit was observed by targeting this mucin.

### 4.6. MUC1-Targeted Therapy

MUC1 is a heavily glycosylated transmembrane glycoprotein that is overexpressed in many carcinomas [83, 84]. MUC1 consists of three domains (a large extracellular motif, a transmembrane motif, and a cytoplasmic tail) [85] and mediates signal transduction events that stimulate the motility, invasion, and metastasis of cancer cells. MUC1 is overexpressed on 90% of early ovarian cancer cell surfaces [83]. In cancer patients, humoral and cellular responses against MUC1 have been detected [86, 87]. Thus, MUC1 has been recognized as a promising molecular target for immunotherapy in patients with ovarian cancer.

The mAb Human Milk Fat Globule 1 (HMFG1) is a murine MoAb that recognizes an epitope localized in the extracellular MUC1 domain.

In a first phase I/II study, Yttrium-90-labeled HMFG1 alone or in combination with Yttrium-90-labeled MoAb AUA1 (directed against an unspecified ovarian cell surface antigen) was intraperitoneally administered to 25 patients with advanced ovarian cancer, who previously had undergone cytoreductive surgery followed by chemotherapy [88]. Fourteen patients had assessable tumour at laparoscopy; none of the three patients with tumour nodules greater than 2 centimetres diameter showed any response to treatment, although one patients experienced resolution of her ascites. One out of ten patients with tumour nodules less than 2 centimetres diameter had a partial response which persisted for one year. Most frequent toxicities consisted in reversible myelosuppression and thrombocytopenia.

An extended study [89] comparing radioimmunotherapy (90Y-labeled HMFG1) after chemotherapy with chemotherapy alone in 45 ovarian cancer patients, disease-free at second-look laparoscopy, found that the active arm had a significantly higher OS (80%) at five-year follow-up, as compared to the control group (50%).

Ten years later, a clinical study [90] carried out on 52 ovarian cancer patients (40% in complete clinical remission and 60% with residual disease) revealed that a single intraperitoneal administration of ^90^Y-radiolabeled HMFG1 could prolong long-term survival, with a 10-year survival rate of 70%.

In 2004, 26 women affected by ovarian cancer received a priming dose of 25 mg of HMFG1 either intravenously (*n* = 10) or intraperitoneally (*n* = 16), followed by 6 intradermal immunizations of HMFG1 in 10% Alhydrogel at 1-month intervals [91]. The 3 dose levels were 0.5 mg, 1 mg, and 5 mg. Thirteen out of 26 patients completed the treatment, while the other patients had clinical disease progression.

ELISA showed that all patients generated measurable anti-idiotypic Ab (Ab2) after 3 immunizations, sustained at 1 month after the final booster. No statistically significant differences were observed in the levels of Ab2 generated by higher or lower booster doses or between patients whose first immunization was administered intravenously or intraperitoneally. 5/13 patients (38%) increased anti-MUC1 levels above 0.015 *μ*g/mL (pretreatment peak). Ab3 (anti-anti-idiotypic Ab) changes for the group as a whole were not statistically significant (*P* = 0.065). Furthermore, anti-MUC1 levels did not correlate with Ab2 levels. Biosensor assay, using the resonant mirror biosensor, showed no difference in the affinity of Ab2 generated by different booster doses of HMFG1. No clinical response was detected in patients with measurable disease, although 1 patient remained without clinical disease for 5 years after completion of vaccination.

The major study investigating safety and efficacy of Y-90-labeled HMFG1 [92] was carried out on 447 women with FIGO stage IC to IV epithelial ovarian cancer in complete remission of disease, surgically assessed through a second-look laparoscopy. In this randomized control study, 224 patients were assigned to receive standard treatment plus a single intraperitoneal infusion of 25 mg Y-90-labeled HMFG1, whereas 223 patients received standard treatment alone. Clinical results showed that there was no significant difference in terms of OS (*P* = 0.4033) and PFS (*P* = 0.4764) between both groups, concordantly with time to serological relapse (CA125 increase) (*P* = 0.3140). However, it was observed [93] that significantly fewer intraperitoneal (*P* < 0.05) and more extraperitoneal (*P* < 0.05) relapses occurred in patients who received Y-90-labeled HMFG1. Furthermore, time to IP recurrence was significantly (*P* = 0.0019) longer and time to extraperitoneal recurrence was significantly shorter (*P* < 0.001) for the active treatment arm. Although serious adverse events occurred with no significant differences in both two arms, hematologic toxicities were more frequent in the Y-90-labeled HMFG1, with a peak incidence after the sixth week of treatment. Serum sample from 208 patients in the active treatment group and 199 patients in the standard treatment group were evaluated for anti-MUC1 IgG [94]. Anti-MUC1 IgG titers ranked significantly (*P* < 0.001) higher in the active treatment group when tested at weeks 4, 8, and 12. A significant difference (*P* < 0.001) in terms of median area under the curve (AUC) between both groups was observed in favour of the active treatment group. A significant higher benefit in OS and disease-free survival (*P* = 0.043 and 0.036, resp.) for patients of the active treatment group with an anti-MUC1 IgG AUC > 13 was shown by multivariate analysis and Kaplan-Mayer analysis.

Recently, a humanized variant of the murine HMFG1, AS1402, has been developed and is now being studied in a phase II trial evaluating the efficacy of the combination of AS1402 with hormonal therapy in postmenopausal women with advanced breast cancer [95]. This humanized antibody could represent a potential treatment agent for patients with ovarian cancer.

### 4.7. Targeting Immunesuppressive CTLA-4: Ipilimumab

Cytotoxic T lymphocyte-associated antigen 4 (CTLA-4) is a surface ligand expressed by activated lymphocytes, which binds to B7-1 and B7-2 ligand expressed upon APC membrane for cell-cycle arrest and attenuation of effector function. Consequently, CTLA-4 acts as a negative regulator of immune response. Ipilimumab is a humanized monoclonal antibody blocking CTLA-4, engineered to contrast the negative immune regulation, thus increasing quantity and duration of the immune effector response against tumor cells. Very recently, the results of significant improvement in overall survival obtained in melanoma setting [96] have led the FDA to approve ipilimumab in the treatment of metastatic melanoma disease (http://www.fda.gov/, FDA approval in August 2010), making the emergence of the key role of CTLA-4 as a therapeutic target in oncologic patients possible. Up to now, only one experience has been carried out with ipilimumab in ovarian cancer.

In 2008 Hodi et al. [96] administered 1 to 11 infusions ipilumimab in 9 patients with stage IV ovarian cancer and in 11 patients with metastatic melanoma. Each dose (3.0 mg/kg over 90 minutes) was administered at 2-3 months interval, with the exception of 1 ovarian cancer who was treated at 3- to 6-month intervals. Eligibility criteria included previous vaccinations with irradiated, autologous tumor cells engineered to secrete GM-CSF (GVAX). The most relevant results involved 1 patient, who achieved a significant fall of CA125 levels several months after the first dose of ipilumimab. The antitumoral response did not involve the generation of anti-CA125 Ab, but the increase in humoral response against NY-ESO-1 was associated with therapeutic effects.

## 5. Monoclonal Antibodies in Cervical Cancer

Currently no treatment with MoAbs has been authorized by FDA for patients with cervical and endometrial malignancies and therefore only experimental results are available.

In cervical cancer patients two molecules are currently investigated as target for MoAbs-specific treatment: VEGF and EGFR.

### 5.1. Vascular Endothelial Growth Factor

In cervical cancer patients VEGF overexpression is associated with tumor progression and poor prognosis [97, 98]; higher VEGF levels appear to correlate with a more advanced disease stage and increased risk of lymph nodes metastasis [99].

Furthermore, it has been shown that higher VEGF expression, as well as increased tumor vascularization, is independent predictors of poor disease and OS [100].

Several studies performed on bevacizumab, used as single agent or in association with other drugs, demonstrated that this antibody is able to delay the progression of cervical cancer [101, 102]. All patients received chemoradiation and at least one other chemotherapy regimen prior to this combination therapy with bevacizumab. After treatment, one patient achieved complete response, one partial response, and two showed disease stabilizations. Furthermore we have encouraging results on phase II multicenter trial to assess the efficacy and tolerability of bevacizumab used as single agent in patients with persistent or recurrent squamous cervical carcinoma.

Approximately, 24% of women benefited from a progression free over 6 months and in 11% of patients a partial response was observed [101]. The only phase III trial with MoAb in cervical cancer is ongoing now [103] (Table 4). Results of this phase III trial are expected both for bevacizumab alone and in combination with chemotherapeutic agents in recurrent/persistent or, mostly, stage IV cervical cancer. In IV stage cervical cancer patients, a real standard of care has not been well established nowadays [104]. Surgery, when technically feasible, intuitively appears as the most direct way to eliminate tumour burden and overcome radio and chemotherapy resistance caused by size in this stage of disease. Unfortunately, several patients affected by large volume disease are considered inoperable. Neoadjuvant chemotherapy (NACT) has demonstrated to increase the proportion of women amenable of surgery and reduce negative pathologic prognostic factors [105, 106]. Severe prognosis is associated with this stage and the fact that these patients are affected by chemo-naive neoplasms makes this setting of women particularly adequate to test new combinations drug that include target therapies.

### 5.2. Epidermal Growth Factor Receptor

EGFR is tyrosine kinase receptor of the family that includes HER2, HER3, and HER4. This receptor mediates cell differentiation and proliferation in both embryonic and adult tissues.

This receptor is overexpressed in approximately 85% of invasive cervical tumours and is associated with higher stages and poor prognosis [107–111]. Blockage experiments of this receptor show that it exerts a positive modulation of adjuvant treatments. In particular, in human tumour xenograft *in vivo* model MoAbs showed synergistic effects with cisplatin and doxorubicin [112] and in human this effect was observed with radiotherapy [57].

Between MoAb directed against EGFR, the ones that are currently studied in cervical cancer patients are cetuximab and matuzumab.

Cetuximab (Table 3) is a chimeric IgG1 mAb that antagonizes normal ligand receptor interactions and therefore disrupts EGFR downstream signaling. The relation between EGFR protein expression and response to mAb is doubtful, as colorectal cancer patients without protein overexpression may respond to cetuximab [113].

Preclinical studies developed on cervical cancer cell lines [114, 115] confirm even in this tumor the results obtained in murine model from Baselga group: both chemo and radiotherapy join of cetuximab coadministration effects but apparently in a less EGFR-dependent way [115]. Less encouraging are clinical results. No PFS and OS benefits have been registered in cervical cancer patients in either advanced, recurrent, metastatic, or pretreated disease [116–119]. However, better outcomes could result from an ongoing clinical trial evaluating the addition of cetuximab to standard treatment in patients with early stages of cervical carcinoma.

## 6. Monoclonal Antibodies in Endometrial Cancer

In endometrial cancer patients only VEGF is currently investigated as target for MoAbs-specific treatment. Therefore, data on the possible role of Bevacizumab in endometrial cancer are still scarce [120, 121].

### 6.1. Vascular Endothelial Growth Factor

VEGF is critical for angiogenesis and tumor progression. Preliminary results from studies conducted with the purpose of evaluating the role of antiangiogenic agents in patients with endometrial cancer are encouraging [121]. A study [120] on recurrent or persistent endometrial cancer with bevacizumab showed 8/53 (15.1%) response rate, with 1 complete response and 7 partial responses. Median PFS was 4.2 months. Median OS was 10.5 months. Several clinical trials are currently ongoing (Table 5).

## 7. Discussion and Conclusion

Monoclonal antibodies have demonstrated to be effective in both hematologic and solid malignancies. This family of antineoplastic agents have several different mechanisms, such as binding soluble ligands, blocking cell receptors, and activating ADCC.

In ovarian cancer, encouraging results have been observed with bevacizumab in first and second line settings, mostly in association to standard chemotherapy regimens. Currently, the primary goal in combining bevacizumab to standard chemotherapy is to test its efficacy in increasing the duration of first remission. Preliminary results of randomised trials carried out with this purpose seem to confirm a benefit in terms of progression-free survival, whereas data regarding overall survival remain currently less clear. Furthermore, on the basis of the Japanese experience [122], the GOG 262 is now testing the association between bevacizumab and paclitaxel in a dose dense front line regimen. The rational of combining bevacizumab to weekly paclitaxel in first line setting dates back to recent evidences showing that this association significantly improves progression-free survival in heavily pretreated recurrent epithelial ovarian cancer [123, 124], thus confirming the role of weekly paclitaxel plus bevacizumab in synergistically inhibiting angiogenesis [125].

As single agent in ovarian cancer palliative setting, cetuximab remains one of the most successful monoclonal antibodies to be employed, with demonstrated and approved high efficacy in the management of ovarian cancer-related malignant ascites. Another promising antibody, in gynecologic oncology, seems to be farletuzumab, targeting the folate receptor which is widely expressed by ovarian cancer cells.

Despite the recognized clinical role of trastuzumab-based therapy in breast cancer, current evidence seems to deny any possible clinical relevance of single-agent trastuzumab-based treatment both in ovarian cancer and in endometrial cancer settings [126]. Studies assessing the association between trastuzumab and standard chemotherapy regiments in these types of gynecological malignancies are required.

Promising results have been currently obtained in ovarian cancer setting by single-agent HMFG1 administration, even if stratification of the results in terms of tumor histology would clarify the most appropriate subset which can mostly benefit from anti-MUC1 vaccination. Clinical evaluation of combining HMFG1 to chemotherapy is strongly needed.

Currently, no antibody has shown a particularly high activity in cervical neoplasm. The high expression rate of EGFR, targeted by cetuximab, makes this monoclonal antibody one of the most studied new drugs, even if some concern has been raised for the tolerability of this drug in previously irradiated patients. In ovarian cancer, this drug revealed no positive clinical benefit, both as single-agent and in association to standard chemotherapy regimens. Furthermore, its combination to chemotherapy seems to enhance the risk of treatment-associated adverse events, thus discouraging future employment of this monoclonal antibody in this setting.

In endometrial cancer some experience has been gained with bevacizumab. Results appear comparable to what observed with nontarget drugs.

Up to now, target therapies have not been investigated in rare gynecological malignancies such as vagina and vulvar cancer.

Target therapies and in particular monoclonal antibodies were introduced in oncology with the expectation of having extremely favorable side effects. On the contrary, hemotoxicity, dermotoxicity, gastrointestinal toxicity, and high rates of thromboembolic events have all been reported. With the exception of bevacizumab, no target therapy has yet shown a clear therapeutic effect in gynecological malignancies.

Target therapies are in their infancy in gynecologic oncology. The magnitude of their clinical impact is yet to be seen. A crucial point that requires further investigations remains to be patient selection and targets identification.

MoAbs are destined to become an important tool in the hands of oncologists that treat neoplasms of the genital tract but, as all other established treatments, they will carry the burden of a learning curve to manage their new side effects.

## Figures and Tables

**Table 1:** FDA-approved MoAbs for cancer patients.

Monoclonal antibody	Target	Approved cancer patients	Mode	Year of introduction
Rituximab	CD20	Non-Hodgkin Lymphoma	Chimeric IgG1	1997
Trastuzumab	ErbB2	Breast	Humanized IgG1	1998
Gemtuzumab-ozogamicin	CD33	Acute myeloid leukemia	Humanized IgG4 + ozogamicin	2000
Alemtuzumab	CD52	Chronic lymphocytic leukemia	Humanized IgG1	2001
Ibritumomab tiuxetan	CD20	Non-Hodgkin's lymphoma	Murine IgG1+Yttrium90	2002
I-Tositumomab	CD20	Non-Hodgkin's lymphoma	Murine IgG2a+iodine-131	2003
Cetuximab	EGFR	Colorectal Head/Neck	Chimeric IgG1	2003
Bevacizumab	VEGF	Colorectal	Humanized IgG1	2004
Panitumumab	EGFR	Colorectal	Humanized IgG2	2006
Ofatumumab	CD20	Chronic Lymphocytic Leukemia	Human IgG1	2009
Ipilimumab	CTLA-4	Late stage melanoma	Human IgG1	2011

**Table 2:** Ongoing treatment studies evaluating MoAb treatment in ovarian cancer patients.

Protocol number	Disease stage	Target therapy	Treatment	Study phase	PI
NCT00565851 (GOG-213)	Recurrent	Bevacizumab	Arm I:	Phase III	Coleman RL, MD
			Carboplatin + Paclitaxel/Docetaxel every 3 weeks		
			Arm II:		
			Carboplatin + Paclitaxel/Docetaxel + Bevacizumab every 3 weeks		

NCT00483782 (ICON 7)	Primary	Bevacizumab	Arm I:	Phase III	Perren TJ, MD
			Carboplatin + Paclitaxel for 6 cycles		
			Arm II:		
			Carboplatin + Paclitaxel + Bev for 6 cycles + Bev for 12 cycles		

NCT00849667	Recurrent	Farletuzumab	Arm I:	Phase III	Morphotek, Inc
			Carboplatin + Taxane + Farletuzumab 1.25 mg/kg		
			Arm II:		
			Carboplatin + Taxane + Farletuzumab 2.5 mg/kg		
			Arm III:		
			Carboplatin + Taxane + Placebo		

NCT00951496	Primary	Bevacizumab	Arm I:	Phase III	Joan L. Walker, MD
			Carboplatin + Paclitaxel + bevacizumab for 6 cycles + bev until progression/recurrence		
			Arm II:		
			Carboplatin + Paclitaxel + bevacizumab for 6 cycles		
			Arm III:		
			Carboplatin + Paclitaxel + bevacizumab for 6 cycles		

NCT00976911	Primary	Bevacizumab	Arm I:	Phase III	Hoffmann-La Roche
			Topotecan + Paclitaxel + liposomal doxorubicin		
			Arm II:		
			Bevacizumab + Topotecan + Paclitaxel + liposomal doxorubicin		

NCT01081262	Primary	Bevacizumab	bevacizumab	Phase III	Martin E. Gore, MD
			capecitabine		
			carboplatin		
			oxaliplatin		
			paclitaxel		
			Procedure: quality-of-life assessment		

NCT01167712	Primary	Bevacizumab	Arm I: Carboplatin + Paclitaxel	Phase III	John K. Chan, MD
			Arm II: Carboplatin + Paclitaxel		

NCT01239732	Primary	Bevacizumab	Arm I: Carboplatin + Bevacizumab + Paclitaxel	Phase III	Hoffmann-La Roche

**Table 3:** Ongoing treatment studies evaluating EGFR MoAbs in cervical cancer patients.

Protocol number	Disease stage	Target therapy	Adjuvant treatment	Study phase	PI
NCT00803062	Stage IVb/	Bevacizumab	Cisplatin/topotecan hydrochloride/paclitaxel	Phase III	Krishnansu Tewari, MD
	recurrent/persistent				
NCT00548418	Recurrent/Persistent	Bevacizumab	Topotecan/cisplatin	Phase II	Janet S Rader, MD

**Table 4:** Ongoing treatment studies evaluating VEGF MoAbs in cervical cancer patients.

Protocol number	Disease stage	Target therapy	Adjuvant treatment	Study phase	PI
NCT00292955	locally advanced/metastatic	Cetuximab	Cisplatin + radiotherapy	Phase II	Linda R. Duska, M.D

NCT00104910	Stages Ib-IVA	Cetuximab	Cisplatin + radiotherapy + brachitherapy	Phase I	John H. Farley, MD

NCT00997009	Advanced/Recurrent	Cetuximab	Paclitaxel + carboplatin	Phase II	Sandro Pignata, MD

NCT00957411	Stages IB-IIIB	Cetuximab	Cisplatin	Phase II	Susan Scholl, MD

NCT01158248	Stages Ib-III	Panitumumab	Cisplatin + Radiotherapy + brachitherapy	Phase II	Alain Zeimet

NCT01301612	Adenocarcinoma	Nimotuzumab	Cisplatin + Radiotherapy + brachitherapy	Phase II	Sergio Lago

**Table 5:** Ongoing treatment studies evaluating MoAbs in endometrial cancer patients.

Protocol number	Disease stage	Target therapy	Adjuvant treatment	Study phase	PI
NCT01010126	Endometrial cancer	Bevacizumab	Temsirolimus	Phase II	Charles Erlichman, MD

NCT00977574	Endometrial cancer	Bevacizumab	Temsirolimus Carboplatin ixabepilone Paclitaxel temsirolimus	Phase II	Carol Aghajanian, MD

NCT01005329	Endometrial cancer	Bevacizumab	Carboplatin Cisplatin	Phase II	Akila Viswanathan, MD
			Paclitaxel radiotherapy		

NCT01367002	Uterine serous	Trastuzumab	Carboplatin Paclitaxel	Phase II	Alessandro D Santin, M.D.

NCT01256268	Endometrial Cancer	Ridaforolimus	Paclitaxel Carboplatin	Phase I	Robert Wenham, M.D.

NCR01244438	Endometrial Cancer with FGFR mutation	FP-1039	/	Phase II	Sarah Thayer

NCT01065246	Epithelial Carcinomas	Catumaxomab		Phase II	Jalid Sehouli, MD
